# Effect of Melatonin on Redox Enzymes Daily Gene Expression in Perirenal and Subcutaneous Adipose Tissue of a Diet Induced Obesity Model

**DOI:** 10.3390/ijms24020960

**Published:** 2023-01-04

**Authors:** Pilar Fernández-Mateos, Pilar Cano-Barquilla, Vanesa Jiménez-Ortega, Leire Virto, Juliana Pérez-Miguelsanz, Ana I. Esquifino

**Affiliations:** 1 Instituto de Investigación Sanitaria del Hospital Clínico San Carlos (IdISSC), 28003 Madrid, Spain; 2 Department of Cellular Biology, Faculty of Medicine, Complutense University, 28040 Madrid, Spain; 3 Department of Biochemistry and Molecular Biology, Faculty of Medicine, Complutense University, 28040 Madrid, Spain; 4 Department of Anatomy and Embryology, Faculty of Optics, Complutense University, 28037 Madrid, Spain; 5 Department of Anatomy and Embryology, Faculty of Medicine, Complutense University, 28040 Madrid, Spain

**Keywords:** obesity, melatonin, redox enzymes, day/night changes, subcutaneous adipose tissue, perirenal adipose tissue, male wistar rats

## Abstract

Increased adiposity is related to oxidative stress, inflammation and metabolic disorders. Our group has shown that melatonin totally or partially prevents the alterations that obesity causes in some neuroendocrine and inflammatory parameters indicative of oxidative stress. This study analyzes the effects of HFD on the relative gene expression of several redox balance enzymes on adult male Wistar rats subcutaneous (SAT) and perirenal adipose tissue (PRAT) and the possible preventive role of melatonin. Three experimental groups were established: control, high fat diet (HFD) and HFD plus 25 μg/mL melatonin in tap water. After 11 weeks, animals were sacrificed at 09:00 a.m. and 01:00 a.m. and PRAT and SAT were collected for selected redox enzymes qRT-PCR. Differential expression of redox enzyme genes, except for SOD^Mn^, GPx and catalase, was observed in the control group as a function of fat depot. HFD causes the disappearance of the temporal changes in the expression of the genes studied in the two fat depots analyzed. PRAT seems to be more sensitive than SAT to increased oxidative stress induced by obesity. Melatonin combined with a HFD intake, partially prevents the effects of the HFD on the gene expression of the redox enzymes. According to our results, melatonin selectively prevents changes in the relative gene expression of redox enzymes in PRAT and SAT of animals fed an HFD.

## 1. Introduction

The World Health Organization (WHO) [1] defines obesity as an excessive accumulation of fat in adipose tissue that can be detrimental to health. Considered as a chronic pathology of multifactorial origin, obesity is in itself a major risk factor for the development of a wide range of metabolic diseases [2,3,4]. Besides, the changes in other parameters of the neuroendocrine and immune systems [5,6], may be responsible of the associates comorbidities described from cardiovascular to renal dysfunctions or inflammatory diseases [7,8,9].

Adipose tissue was shown to synthesize and secrete adipokines that are pertinent to glucose and lipid homeostasis, as well as inflammation. Importantly, the obesity-induced adipose tissue expansion stimulates signals capable of triggering an inflammatory response. These inflammatory manifestations of obese adipose tissue have been linked to insulin resistance, metabolic syndrome, and type 2 diabetes. A number of experimental models of obesity from genetic [10] to high-fat diets administration [11,12] have been developed, to analyze this syndrome [13,14].

The accumulation of subcutaneous adipose tissue favors a greater systemic tolerance to glucose and has effects on metabolism [15]. However, in the early stages of obesity, subcutaneous adipose tissue (SAT) reduce its capacity to store lipids, so that these would be deposited in the form of visceral fat [3], thus leading to a generalized inflammation as has being suggested by previous studies analyzing plasma levels of cytokines in obesity [16,17,18,19]. Metabolic disorders, characterized by AT inflammation and accumulation around organs may eventually induce organ dysfunction through a direct local mechanism. Perirenal adipose tissue (PRAT), surrounding the kidney, influences renal function and metabolism. In this regard, PRAT emerged as an independent risk factor for chronic kidney disease (CKD) and is even correlated with CVD [20]

In addition, the expanded and inflamed fat generates free radicals that fulfil various pathological functions and act as signaling molecules [2].

The elimination or neutralization of reactive oxygen species ROS by an antioxidant system, plays an important role in obesity and its comorbidities [21]. ROS decrease adipogenesis favoring to hypertrophy of adipocytes and subsequently alters adipokines secretion and increase macrophage infiltration [22].

Antioxidants act as free radical scavengers and can prevent oxidative reactions that lead to various diseases [23]. The antioxidant defense system includes endogenous (enzymatic and nonenzymatic) and exogenous (dietary) antioxidants that interact in establishing redox homeostasis in the body [24]. Endogenous antioxidants, which are products of the body’s metabolism, may be enzymatic or nonenzymatic compounds localized generally in the cytoplasm and diverse cell organelles [25]. In eukaryotes, various antioxidant enzymes, for instance, superoxide dismutase (SOD), catalase (CAT), and some peroxidases, transform ROS into more stable molecules (e.g., water and O_2_) via complex cascade of reactions. One of the most effective intracellular enzymatic antioxidants is SOD. SOD catalyzes the dismutation of O_2_^•−^ to H_2_O_2_, decreasing the amount of O_2_^•−^ and thereby lowering the formation of ONOO− [26,27]. Other important enzymatic antioxidants include CAT, glutathione peroxidase (GPx) and glutathione reductase (GRed). These enzymes neutralize hydrogen peroxide, yielding water (CAT, GPx) and oxygen molecule (CAT). GPx can be found in many subcellular compartments including the mitochondria and nucleus depending on the family member [28]. GPx uses reduced glutathione (GSH) as a substrate to transfer electrons to H_2_O_2_ (and other peroxides), thereby converting it into two molecules of water [29]. When hydrogen peroxide is metabolized by glutathione peroxidase, reduced glutathione is oxidized to glutathione disulfide (GSSG) which is converted back to GSH by the enzyme GRed [30,31].

On the other hand, the circadian system synchronizes physiology and behavior with a 24-hour environmental cycle and optimizes energy balance [32]. Photoperiod-induced changes in oxidative stress biomarkers have been described indicating the existence of a daily rhythm of gene expression in rat antioxidants enzymes [33,34]. In this regard, disruptors that can alter the daily gene expression of oxidative stress enzymes have also been described [35].

Our group previously published that obesity is a disruptor of the neuroendocrine circadian system [5,11,36]. Specifically, obesity modifies the circadian pattern of circulating adipokines, metabolic hormones, biochemical parameters of mild inflammation and the expression of hypothalamic genes involved in the regulation of food intake [16,37].

Melatonin completes the antioxidant system as it is ubiquitous and has membrane receptors distributed in peripheral tissues [38]. Melatonin is present in adequate concentrations in cells and reacts with a wide variety of free radicals that have a short half-life due to its high reactivity. It is produced in situ in a wide variety of tissues and can also be acquired from the diet as it is present in a large variety of foods. In addition, melatonin is an ideal natural antioxidant because its toxicity is minimal as has been demonstrated in numerous in vivo studies in animals and humans [39,40]. The association between obesity, mild inflammation and oxidative stress was suggested by the observations that lower levels of antioxidant systems are present in obese subjects [41].

Melatonin, in addition to being an effective antioxidant, is a chronobiotic hormone. Changes in photoperiod duration are converted into neuroendocrine signals by melatonin (i.e., regulation of energy metabolism, especially in glucose and lipid metabolism). Increasing evidence demonstrates a link between melatonin and obesity [42].

Our group has previously shown that melatonin administered from day 1 of the high-fat diet intake partially prevent the disruptive effects of obesity on the neuroendocrine system [37] and normalizes some clinical and biochemical parameters of mild inflammation in diet-induced metabolic syndrome in rats [16]. In particular melatonin decreased the augmented circulating levels of IL-1b, IL-6, TNF-a, IFN-c, and CRP seen in obese rats and restored the depressed levels of IL-4 and IL-10 and avoid the weight increase observed in obese animals and normalized glucose levels [36]. In addition, protective effect of melatonin were previously described in other experimental obesity animal models [42,43,44] and generically in other pathophysiological situations [35,45,46].

For all these reasons, the present study was designed to analyze the effects of photoperiod on the relative 09:00 a.m. vs. 01:00 a.m. gene expression of enzymes involved in redox balance of two physiologically different fat depots: SAT and PRAT (as visceral fat) in adult male Wistar rats. The second part of the study is to analyze the effect of a high fat diet as a possible disruptor of the adipose tissue circadian system and which of the two fat depots is more sensitive to the effect of the high fat diet.

Finally, it will be assessed the possible preventive effect of melatonin on daily gene expression of redox enzymes in PRAT and SAT of male Wistar rats fed a control or a high fat diet.

## 2. Results

### 2.1. Relative Gene Expression of Heme oxygenase 1 (HO-1) and Heme oxygenase 2 (HO-2) Enzymes 

***Subcutaneous adipose tissue***

**Figure 1:**
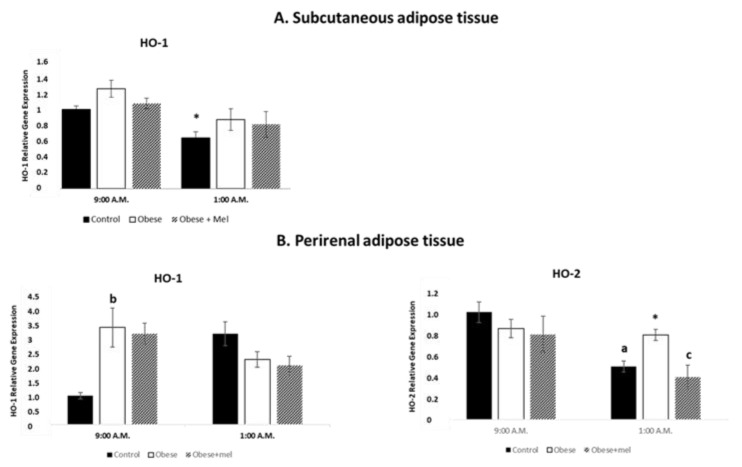
Relative gene expression of HO1 enzyme in subcutaneous (**A**) and perirenal (**B**) adipose tissue and HO2 in perirenal adipose tissue of male Wistar rats at two different times (9:00 a.m. and 1:00 a.m.). Each value represents the mean and s.e.m. (n = 5). The letter indicates significant differences between diets (control or high fat diet) at the same schedule (^a^ *p* < 0.05 vs. control; ^b^ *p* < 0.001 vs. control group of animals; ^c^ *p* < 0.05 vs. obese group of animals) and the asterisk (* *p* < 0.05) indicates significant differences between schedules in each experimental group.

Heme oxigenases play a pivotal role in regulating redox homeostasis in virtue of their anti-inflammatory, antioxidant, and anti-apoptotic properties. Heme degradation products are all biologically active molecules mostly implicated in tissue redox homeostasis and are generated under a multitude of pathological conditions. Considering that previous parameters studied with our experimental model varies depending on the presence or absence of light, we performed the experiment considering a key time of the light period (09:00 a.m.) and a key time of the dark period (01:00 a.m.). Figure 1A shows the effect of HFD on the relative gene expression of Heme oxygenase 1 (HO-1) and Heme oxygenase 2 (HO-2) enzymes. In control group, a decrease in the relative gene expression of HO-1 at 01:00 a.m. (ANOVA *p* < 0.015) was evaluated as compared to the values found at 09:00 a.m. In obese rats, no significant differences were found between the time points analyzed.

On the other hand, in our experiment we aimed to analyze whether the increase in adiposity generated by the administration of a high-fat diet affected the redox system of subcutaneous adipose tissue considered as an example of a fat depot less dynamic than visceral adipose tissue. In view of our results, HFD does not significantly alter HO-1 gene expression at any of the time points studied.

In addition to photoperiod and diet, in previous studies, our group has described that the concomitant administration of melatonin with a high-fat diet had a preventive effect on the increase in rat weight gain in absence of any significant effect of melatonin on food intake. Besides, the nocturnal melatonin peak is drastically reduced in obese animals as has been earlier described. For this reason, we administered exogenous melatonin to the animals fed high-fat diet to find out if melatonin would have a potential preventive effect on redox enzymes gene expression. In view of our results melatonin administration did not modify HO-1 values in obese rats at any of the time points analyzed.

Surprisingly, HO-2 was not detected in SAT of control rats at any of the times tested. Likewise, this enzyme was not detected either in obese rats or in obese rats treated with melatonin.

***Perirenal adipose tissue***

Along with the same line of thought, in this case we have chosen as a representative of visceral adipose tissue, the perirenal fat due to its implications with renal pathologies. Figure 1B shows the effect of HFD on the relative gene expression of HO-1 and HO-2 enzymes in PRAT of male Wistar rats, at a representative time point of the light (09:00 a.m.) and dark (01:00 a.m.) photoperiod. Regarding to HO-1 we have demonstrated that in the perirenal tissue of the control group, there was an increase in HO-1 gene expression at 01:00 (ANOVA *p* < 0.025) compared to the values obtained at 09:00. However, there were no differences in the relative HO-1 gene expression values in the obese rats according to the time of day analyzed. If we consider the effect of diet in each time analyzed separately it can be seen an increase in the expression of HO-1 gene in obese rats (ANOVA *p* < 0.001) at 09:00 a.m. but not at 01:00 a.m.

Regarding the effect of exogenous melatonin administration on the HO-1 relative gene expression of animals fed a high-fat diet, there was an increase in the expression of HO-1 gene expression by the administration of the high fat diet in combination with melatonin (ANOVA *p* < 0.001) at 09:00 a.m. Administration of melatonin did not modify the values obtained in obese rats in any of the analyzed schedules.

In contrast to SAT, in PRAT, HO-2 gene expression is observed in all groups studied. If we consider the effects that are observed in the expression of this gene in the presence or absence of the light at 01:00 a.m., there was a decrease in the relative expression of HO-2 gene (ANOVA *p* < 0.002) when compared with that obtained at 09:00 a.m. in the control group. With this design, the administration of a high fat diet abolished the hourly differences in HO-2 gene expression.

Considering the effects of the diet in each schedule separately, it is noteworthy that, at 01:00 a.m., there was an increase in HO-2 gene expression (ANOVA *p* < 0.047) in the obese group, compared with the values obtained in the control group.

The administration of melatonin along with the high fat diet, did not modify the values obtained in obese rats at 09:00 a.m. Moreover, Melatonin administration prevented the effect of obesity on HO-2 gene expression (ANOVA *p* < 0.009 vs. obese group) at 01:00 a.m.

### 2.2. Relative Gene Expression of Superoxide Dismutase Copper/Zinc (SOD^Cu/Zn^) and the Superoxide Dismutase Manganese (SOD^Mn^) Enzymes 

***Subcutaneous adipose tissue***

**Figure 2:**
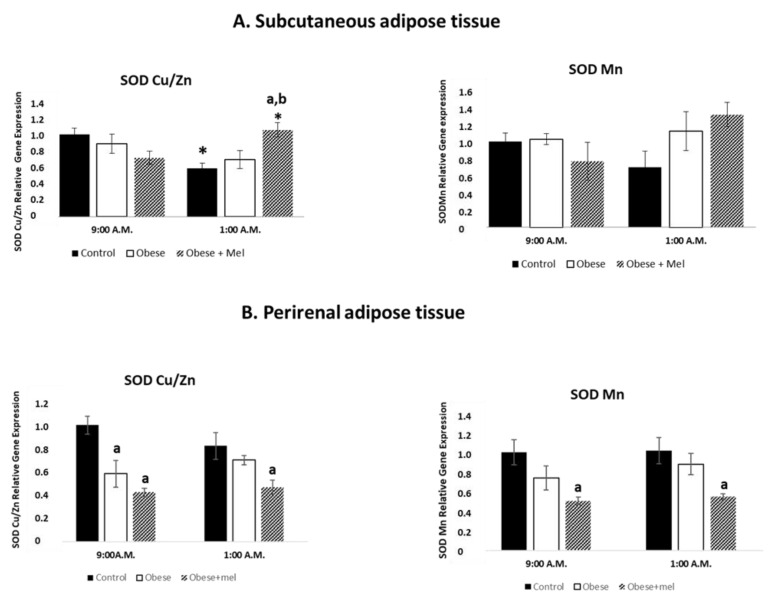
Relative gene expression of SOD^Cu/Zn^ and SOD^Mn^ enzymes in subcutaneous (**A**) and perirenal (**B**) adipose tissue of male Wistar rats at two different schedules (09:00 a.m. and 01:00 a.m.). Each value represents the mean and s.e.m. (n = 5). The letter indicates significant differences between diets (control or high fat diet) at the same schedule (^a^ *p* <0.05 vs. control; ^b^ *p* < 0.001 vs. obese group of animals) and the asterisk (* *p* < 0.05) indicates significant differences between schedules in each experimental group.

Measurements of antioxidant status that include antioxidant enzymes (e.g., SOD) can also be utilized as oxidative stress biomarkers. For this reason, using the same experimental protocol, we evaluated the effects of photoperiod, type of diet and exogenous melatonin administration on the superoxide dismutase Copper/Zinc (SOD^Cu/Zn^) and Superoxide Dismutase Manganese (SOD^Mn^) relative gene expression. Figure 2A shows these effects in SAT of male Wistar rats, at two representative time points around the clock: one of the lights (09:00 a.m.) and another one of the dark (01:00 a.m.) photoperiod.

In the control group, there were hourly differences in the SOD^Cu/Zn^ gene as happened for the other enzymes before mentioned. In fact, there was a significant decrease in the relative expression of the SOD^Cu/Zn^ gene at 01:00 a.m. (ANOVA *p* < 0.006) as compared to the values found at 09:00 a.m. in the subcutaneous tissue. However, in the obese group there are no variations in the levels of SOD^Cu/Zn^ gene expression at the times studied. It is of interest, that melatonin increased the expression of this gene at 01:00 a.m. when compared with the values observed in the control group. (ANOVA *p* < 0.05).

If we consider the effect of the diet on each timetable separately, in the obese group there are no variations in the levels of SOD^Cu/Zn^ gene expression at the times studied.

When exogenous melatonin is administered to animals fed HFD, at 09:00 a.m. melatonin administration showed no differences compared to control or HFD fed rats, at 01: 00 a.m. melatonin significantly increases SOD^Cu/Zn^ gene expression both when compared to control animals (ANOVA *p* < 0.05) and when compared to animals fed an HFD (ANOVA *p* < 0.001)

Surprisingly, SOD^Mn^ gene expression values did not show significant differences in any of the groups studied or at any of the schedule time.

***Perirenal adipose tissue***

The Figure 2B shows the effect of HFD on the relative gene expression of SOD^Cu/Zn^ and SOD^Mn^ enzymes in PRAT of male Wistar rats, at two representative time points around the clock: one of the lights (09:00 h) and another one of the dark (01:00 h) photoperiod.

With the same experimental design as in previous enzymes, the SOD^Cu/Zn^ gene expression values in the control group did not show significant differences in the studied schedules. There were also no significant differences in function of the time points analyzed in either the obese group or the melatonin-treated obese group.

Regarding the effects derived from HFD intake in each time point separately, it is of interest that at 09:00 a.m. there was a decrease in SOD^Cu/Zn^ gene expression in obese animals when compared to the control group (ANOVA *p* < 0.0018).

Surprisingly, the administration of melatonin along with the high-fat diet further decrease the SOD^Cu/Zn^ gene expression when compared to the control group (ANOVA *p* < 0.004) at 09:00 a.m.

Interestingly, at 01:00 a.m., melatonin administered together with the high fat diet significantly decreased SOD^Cu/Zn^ gene expression as compared to the control group (ANOVA *p* < 0.018) in the perirenal fat.

Regarding to photoperiod effects on the SOD^Mn^ gene expression as it was shown for SOD^Cu/Zn^, the values of the expression of SOD^Mn^ gene showed no significant differences as a function of the times analyzed in the three groups studied.

When assessing the effects of diet regardless of the time of day studied, results showed that animals fed a HFD showed no significant differences on the expression values of SOD/Mn gene expression compared to control group in any of the scheduled studied.

The results obtained on SOD^Mn^ gene expression, when melatonin is administered with the HFD, showed that the expression of this gene decreased only in the group treated simultaneously with the high fat diet and melatonin, with respect to the control group, both at 09:00 a.m. (ANOVA *p* < 0.046) and at 01:00 a.m. (ANOVA *p* < 0.018).

### 2.3. Relative Gene Expression of Glutathione Peroxidase (GPx) and Glutathione Reductase (GRed) Enzymes 

***Subcutaneous adipose tissue***

**Figure 3:**
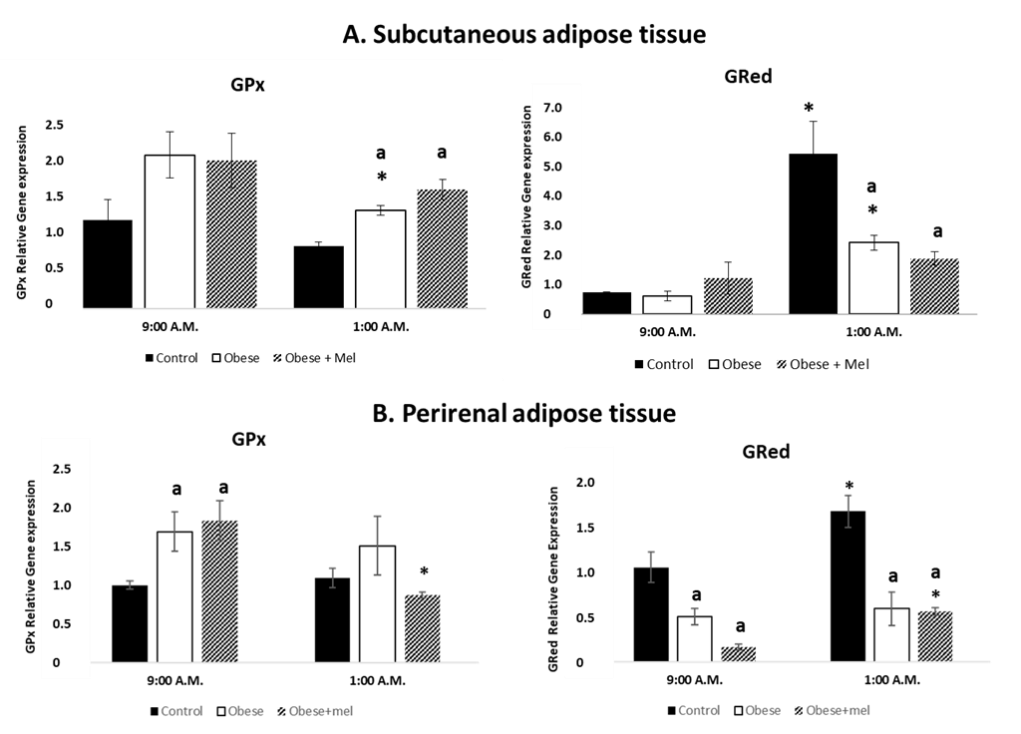
Relative gene expression of GPx and GRed enzymes in subcutaneous (**A**) and perirenal (**B**) adipose tissue of male Wistar rats at two different times (9:00 a.m. and 1:00 a.m.). Each value represents the mean and s.e.m. (n = 5). (**A**) The letter indicates significant differences between diets (control or high fat diet) at the same schedule (^a^ *p* < 0.05 vs. control) and the asterisk (* *p* < 0.05) indicates significant differences between schedules in each experimental group. (**B**) The letter indicates significant differences between diets (control or high fat diet) at the same schedule: GPx (^a^ *p* < 0.05 vs. control); GRed 09h00 a.m. (^a^ *p* < 0.05 vs. control) GRed 01:00 a.m. (^a^ *p* < 0.001 vs. control). The asterisk indicates significant differences between schedules in each experimental group: GPx (* *p* < 0.006); Control GRed (* *p* < 0.05) HFD+Mel (* *p* < 0.001).

The ratio of reduced to oxidized glutathione (GSH/GSSG) represents a dynamic balance between oxidants and antioxidants. For this reason, an indirect way to observe the possible variation of this parameter is to evaluate the effect of photoperiod, diet, and melatonin on the relative gene expression of GPx and GRed. These enzymes oversee maintaining this balance. Figure 3A shows the effect of HFD on the relative gene expression of GPx and GRed enzymes in SAT of male Wistar rats, at two representative time points around the clock: one of the lights (09:00 a.m.) and another one of the dark (01:00 a.m.) photoperiod.

Unexpectedly, control group, showed no significant differences between 09:00 a.m. and 01:00 a.m. values on GPx gene expression. Interestingly, we found day/night differences in the high fat diet treated group with a decrease in their expression at 01:00 a.m. (ANOVA *p* < 0.045).

When we analyzed the effects of the diet in each of the studied schedules, we only found a significant increase in GPx gene expression at 01:00 a.m. (ANOVA *p* < 0.001).

Melatonin has no effects at 09:00 a.m. but increased significantly GPx gene expression at 01:00 a.m. compared to control group (ANOVA *p* < 0.005).

If we focus on GRed and analyze the effects of photoperiod on the variations of its relative gene expression, as expected in SAT, GRed gene expression in the control group was significantly increased at 01:00 a.m. (ANOVA *p* < 0.035) as compared to 09:00 a.m.

Although smaller than in the control group, we also found an increase in GRed gene expression in the high-fat group at 01:00 a.m. (ANOVA *p* < 0.05).

When we analyzed the effects of the diet in each of the studied schedules, we observed that HFD exerts no effects on GRed expression at 09:00 a.m. Interestingly, at 01:00 a.m. we found a decrease in GRed gene expression in animals fed high-fat diet alone (ANOVA *p* < 0.03) compared to control animals.

The administration of melatonin along with HFD decreased GRed relative gene expression at 01:00 a.m. (ANOVA *p* < 0.044) compared to control animals. Melatonin did not affect GRed relative gene expression at 09:00 a.m.

***Perirenal adipose tissue***

With the same experimental design, Figure 3B shows the effect of HFD on the relative gene expression of GPx and GRed enzymes in PRAT of male Wistar rats, at two representative time points around the clock: one of the lights (09:00 a.m.) and another one of the dark (01:00 a.m.) photoperiod. As happened in SAT, in PRAT we found no differences between GPx gene expression values at the time points analyzed in control group. Interestingly, in PRAT there was a decrease in GPx gene expression in animals fed HFD combined with the melatonin administration at 01:00 a.m. compared to the same group at 09:00 a.m. (ANOVA *p* < 0.006).

With respect to the effect of diet on GPx relative gene expression we found in PRAT, a significant increase in GPx gene expression at 09:00 a.m. in animals fed HFD (ANOVA *p* < 0.024).

Exogenous melatonin increased significantly GPx gene expression at 09:00 a.m. as compared to control group (ANOVA *p* < 0.036).

The presence or absence of light also has effects on relative GRed gene expression. As happened in SAT, in PRAT, we found in control group an increase in the GRed gene expression at 01:00 a.m. as compared to the 09:00 a.m. values (ANOVA *p* < 0.035). However, in animals fed the high-fat diet, we found no differences in GRed gene expression in the schedules studied. Besides, GRed gene expression was significantly increased at 01:00 a.m. (ANOVA *p* < 0.001) as compared to 09:00 a.m., in the group fed the high fat diet combined with a melatonin treatment.

On the other hand, HFD intake decreased GRed relative gene expression levels at both 01:00 a.m. and 09:00 a.m., (ANOVA *p* < 0.019, at 09:00 a.m. and ANOVA *p* < 0.001 at 01:00 a.m.) compared to control group (standard diet).

Exogenous administration of melatonin to HFD-fed animals decreased GRed relative gene expression at both point schedules (ANOVA *p* < 0.002).

### 2.4. Relative Gene Expression of Catalase Enzyme 

***Subcutaneous adipose tissue***

**Figure 4:**
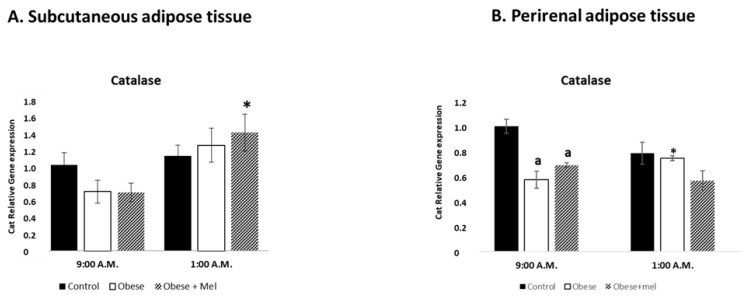
Relative gene expression of Catalase enzyme in subcutaneous (**A**) and perirenal (**B**) adipose tissue of male Wistar rats at two different times (9:00 a.m. and 1:00 a.m.). Each value represents the mean and s.e.m. (n = 5). The letter indicates significant differences between diets (control or high fat diet) at the same schedule (^a^ *p* < 0.05 vs. control) and the asterisk (* *p* < 0.05) indicates significant differences between schedules in each experimental group.

Catalase is one of the crucial antioxidant enzymes that mitigates oxidative stress to a considerable extent by destroying cellular hydrogen peroxide to produce water and oxygen. Deficiency or malfunction of catalase is postulated to be related to the pathogenesis of many degenerative diseases. For this reason, using the same experimental protocol, we evaluated the effects of photoperiod, type of diet and exogenous melatonin administration on Catalase relative gene expression in male Wistar rat SAT at two representative time points around the clock: one of the lights (09:00 a.m.) and another one of the dark (01:00 a.m.) photoperiod (Figure 4A). In SAT we only found a significant increase in catalase gene expression at 01:00 a.m. in the group fed high fat diet combined with a melatonin treatment, as compared with the values obtained at 09:00 a.m. in the same group (ANOVA *p* < 0.033).

Regarding to the effect of diet on catalase relative gene expression, HFD alone or combined with melatonin had no effect compared to the control group in any of the scheduled studied.

***Perirenal adipose tissue***

With the same experimental design, we evaluated the same parameters in PRAT as a visceral adipose tissue (Figure 4B). Regarding photoperiod effects and unexpectedly, in PRAT, catalase relative gene expression, in the control group, showed no significant differences between 09:00 a.m. and 01:00 a.m. values. Interestingly, we found day/night differences in the high fat diet treated group with an increase in their expression at 01:00 a.m. (ANOVA *p* < 0.03).

Interestingly, at 09:00 a.m. we found a decrease in catalase gene expression in animals fed the high fat alone (*p* < 0.001) or combined with melatonin treatment (*p* < 0.005), compared to control animals.

### 2.5. Comparison of the Relative Gene Expression of Each Redox Enzyme between the Two Adipose Fat Depots (PRAT vs. SAT) at the Same Schedule

There are differences between adipose tissue present in subcutaneous areas and visceral adipose tissue. Subcutaneous fat accumulation represents the normal physiological buffer for excess energy intake with limited energy expenditure. It acts as a metabolic sink where excess free fatty acids (FFAs) and glycerol are stored as triglycerides (TGs) in adipocytes. When the storage capacity of SAT is exceeded or its ability to generate new adipocytes is impaired fat begins to accumulate in areas outside the subcutaneous tissue. Visceral adipose tissue (VAT) like PRAT, compared to SAT, is more cellular, vascular, innervated and contains a larger number of inflammatory and immune cells. VAT adipocytes are more metabolically active, more sensitive to lipolysis and more insulin-resistant than SAT adipocytes. VAT has a greater capacity to generate free fatty acids and to uptake glucose than SAT and is more sensitive to adrenergic stimulation. On the contrary, SAT is more avid in absorption of circulating free fatty acids and triglycerides. SAT may be considered as a less sensitive adipose tissue than a visceral fat like PRAT.

For all these reasons, we wanted to evaluate, in an experimental model of obesity induced by a HFD intake, which one of the two adipose tissues SAT, or PRAT was more sensitive to changes in photoperiod and metabolic disruptors like HFD.

On the other hand, we wanted to assess whether the administration of exogenous melatonin simultaneously with the HFD had any protective effect, in any of these adipose tissues, against the expected variations on the relative gene expression of redox system enzymes.

With this line of thought and the values normalized with the 09:00 a.m. controls in both fat depots, we have found that in obese animals, HO1 relative gene expression is significantly increased in PRAT regardless of whether they consumed HFD alone or simultaneously with melatonin.

At 01:00 a.m. the relative gene expression of HO1 was significantly increased in PRAT regardless of the diet fed or melatonin treatment.

HO2 is differentially expressed in both adipose tissues. It is only expressed in PRAT.

At 09:00 a.m. a significant decrease in SOD^Cu/Zn^ gene expression is observed in PRAT of HFD with melatonin fed animals compared to the expression of the enzyme in SAT. The same occurs in dark photoperiod 01:00 a.m.

Regarding SOD^Mn^, during the light photoperiod (09:00 a.m.) the relative gene expression levels of the enzyme are similar in both adipose tissues. However, in the dark phase of the photoperiod there is a significant increase in SOD^Mn^ relative gene expression in PRAT of control animals and a significant decrease in obese animals treated with melatonin.

At 09:00 a.m., the relative gene expression of GPx and GRed are similar in both fats regardless of the diet or melatonin treatment. Nevertheless, at 01:00 a.m., a significant decrease in GPx gene expression is observed in PRAT of obese +MT while GRed gene expression is lower in PRAT of both control and obese animals.

At 09:00 a.m., the relative gene expression levels of Catalase are similar in both adipose tissues regardless of the diet. However, at 01:00 a.m., Catalase relative gene expression are significantly decreased in PRAT of all groups of animals regardless of the diet (Table 1).

## 3. Discussion

Obesity is a heterogeneous disorder. Obese individuals vary in their body fat dis-tribution, their metabolic profile and degree of associated cardiovascular and metabolic risk. Increased adiposity is related to oxidative stress, inflammation and metabolic disor-ders, but this fact does not affect the different fatty deposits equally.

All the above-mentioned data suggest that all the enzymes studied, except for Gx and catalase, showed differential changes in their gene expression as a function of time and tissue analyzed in the control group. The administration of a high-fat diet causes the disappearance of the temporal changes in the expression of the genes studied in the two fat depots analyzed, and previously described in the control group. Melatonin administration combined with a high-fat diet administration, partially prevents the effects of a high fat diet on the gene expression of the enzymes studied.

Hourly differences in gene expression of HO1 and 2, SOD^Cu/Zn^, GRed enzymes indicate the existence of circadian variations in their availability as in other tissues [47]. These differences agreed with the metabolic changes around the clock, previously demonstrated by our group [11,16,36].

Although we did not detect hourly differences in the other enzymes studied, we cannot rule out the existence of variations throughout the day at other times of the day not analyzed in this study.

We found for the first time, that the constitutive HO enzyme is not expressed in the subcutaneous adipose tissue. However, the inducible enzyme is present in perirenal fat, which would indicate a specific handling of the heme group in each fat [47,48]. On the other hand, the results obtained in the perirenal fat confirm the presence of both constitutive and inducible HO enzymes in all groups analyzed, thus indicating the existence of a specific mechanism in each fat depot to manage heme derivates.

The HO system engages with other systems to mitigate the deleterious effects of oxidative stress in obesity and cardiovascular disease (CVD). Other studies indicate that HO1/HO2 protein expression and HO activity have several important roles in hemostasis and ROS-dependent perturbations associated with metabolic syndrome [49]. HO1 protects tissue during inflammatory stress in obesity through the degradation of pro-oxidant heme and the production of carbon monoxide (CO) and bilirubin, both of which have anti-inflammatory and anti-apoptotic properties. In contrast, repression of HO1 is associated with increases of cellular heme and inflammatory conditions including hypertension, stroke and atherosclerosis [49].

HO1, as the only enzyme that degrades the pro-oxidant heme and generates antioxidant products reduces renal oxidative stress and inflammation [50] and glomerular injury [45]. For this reason, it is not surprising that HO1 gene expression levels are increased in the fat surrounding the kidney in obese animals.

Importantly, the time differences in HO gene expression disappeared in animals fed the high-fat diet, indicating that this type of diet may affect both the circadian clock that regulates daily metabolism and the hypothalamic feeding behavior regulatory center [37].

We note that melatonin administration reverses the effects of the high-fat diet on HO2 enzyme only in perirenal fat at 01:00 a.m., suggesting a selective effect of melatonin on heme oxygenase activity. This selective effect of melatonin has been demonstrated by analyzing other parameters [16,36] or by analyzing the activity of the mediobasal hypothalamus using other experimental designs [35,51].

It is interesting to note that although the expression of SOD^Cu/Zn^ (cytoplasm), in the adipose subcutaneous tissue, follows the same pattern as that of HO, confirming previous work [34,35,46], SOD^Mn^ (mitochondria) does not present hourly variations although they have been reported in other tissues [52]. The latter could be due to the different localization of the two enzymes and the fact that in the mitochondria the generation of radicals is constant due to the presence of the respiratory chain. In fact, mitochondria represent both a source of free radicals due to the electron transport chain but also producers of antioxidant enzymes, thus representing a key organelle in determining the cellular redox state [53].

Melatonin may be synthesized by mitochondria, a capacity that was inherited from bacteria, the precursors of mitochondria. As a result, all cells with mitochondria likely have the capacity to produce melatonin. On the other hand, melatonin is a potent protector of mitochondria. In addition to mitochondrial protection, melatonin also influences mitochondrial dynamics. The daily oscillations of mitochondrial functions as well as the morphology seem to fit well with the melatonin circadian rhythm. Melatonin reduces mitochondrial fission and increases their fusion, thereby preserving their normal function. Recently, it has been reported that melatonin modified mitophagy by either the enhancement or the reduction in this process, depending on conditions and cell types [54].

As expected, in animals fed the high-fat diet the hourly differences in the expression of the superoxide dismutase gene, in the adipose subcutaneous fat, disappeared. These results confirm previous work in the literature [5,9,37]. Furthermore, we emphasize that the excess of concentration of both saturated and unsaturated fatty acids in adipose tissue, in this experimental condition, is associated with an increase in the production of free radicals that alter mitochondrial function and reduce the antioxidant capacity [18]. The administration of melatonin combined with the high-fat diet produced a very marked increase in the gene expression of superoxide dismutase at 01:00 a.m., as would be expected due to the maximum production of free radicals due to an increase in the metabolism, during the activity phase and also because melatonin reaches its maximum level of secretion at this time [11,55,56].

On the other hand, in the perirenal fat, the relative gene expression of SOD^Cu/Zn^, during the light period decreased in animals fed a fat diet. These results confirm that the enzymatic activity of these antioxidant enzymes is lower in adipose tissue during obesity [56]. Increased adiposity in the different fat depots increases the concentration of reactive oxygen species and decreases the individual’s antioxidant capacity, which favors the development of pathologies [19].

**Figure 5:**
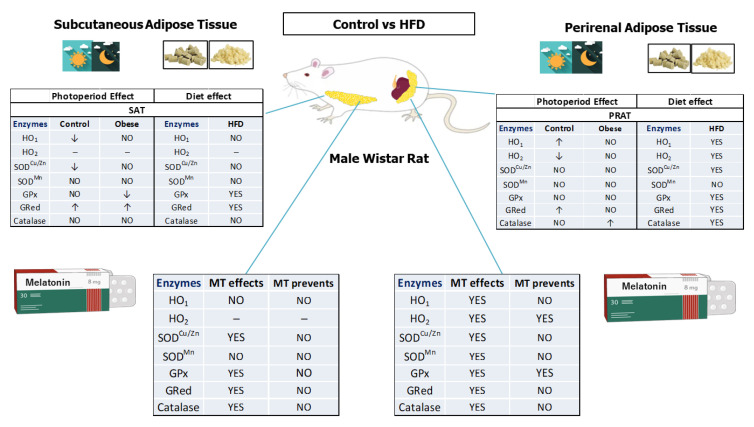
Graphical abstract including effects of photoperiod, diet, and possible preventive effects of Melatonin on 09:00 a.m. vs. 01:00 a.m. gene expression of redox enzymes in PRAT and SAT of male Wistar rats fed a control or a high-fat diet. Arrows indicate whether the relative gene expression of each enzyme is increased (↑) or reduced (↓) comparing same adipose tissue, group, and diet but different schedule.

Surprisingly, the administration of melatonin along with the high fat diet did not prevent the observed changes in SOD expression at least in the schedules studied (Figure 5).

This results may explain recent studies showing an interaction between perirenal fat and kidney function [57].

In the control group, it is interesting to note that in the two fat depots studied, no differences in GPx expression were found in the schedules studied. These differences are present for GRed. The differences may be due to the increased nocturnal metabolic activity.

Surprisingly, in the subcutaneous adipose tissue of animals fed the high-fat diet, significant hourly differences in the expression of the GPx gene appear while the differences observed in the expression of the GRed gene are maintained, albeit to a lesser extent. The data observed in obese rats indicate the existence of a misbalance in these activities, thus leading to a decreased redox activity [58,59].

The changes observed in the expression of GPx and GRed genes in subcutaneous fat may indicate that the dose of melatonin used in this study, which was effective in the central nervous system [36], is not efficient for peripheral tissues and could be used at higher doses to restore antioxidant capacity [60].

In the perirenal fat ingestion of a high-fat diet, regardless of whether the animals were treated with melatonin, significantly increases in the relative gene expression of GPx, during the light phase, while GRed relative gene expression significantly decreases in the two representative schedules of the 24-h cycle, the data may be explained considering that when adiposity increases, the GPx enzyme has a lower affinity for H_2_O_2_ [38]. Maybe, other antioxidant enzymes such as catalase participate in the catabolism of this reactive oxygen species [56]. Furthermore, it has been reported that the enzymatic activity of GRed decreases in adipose tissue of obese animals, which favors the accumulation of free radicals and/or glutathione depletion [61]. In addition, chronic inflammatory state, observed in obesity, decreases the relative gene expression of the enzyme glutathione reductase, which would affect the reduction in oxidized glutathione. These results are similar to other studies in the prefrontal cortex of rats subjected to high oxidative stress [62].

On the other hand, melatonin administration does not prevent the effects of high-fat diet intake on the relative gene expression of GPx and GRed enzymes in perirenal fat. These data differ from results obtained in other tissues such as the hippocampus, where melatonin upregulates GPx enzyme activity in obese animals [51].

Regardless Catalase in control group, this study shows that there were no differences between the two studied schedules in the expression of this gene in SAT. Obesity was not able to modify its expression in subcutaneous adipose tissue, as might be expected considering the data described in obese patients in the visceral fat [9,63]. The effect of melatonin at 01:00 a.m. increased the expression of this enzyme, comparing with 09:00 a.m., confirms previous works from the literature [64,65].

Regarding perirenal adipose tissue as happened for GRed, obesity induced day/night differences in the expression of Catalase with higher levels at night, as would be expected in the active phase of the rat. Our results show that a high-fat diet significantly decreases catalase gene expression at 09:00 a.m., which could contribute to the development of oxidative stress in visceral fat tissue [56]. Our results confirm the studies of Luo et al. [61], who describe that oxidative damage increases the ROS production and reduces catalase gene expression and antioxidant enzyme activity in the kidney. However, other studies have reported that, under conditions of oxidative stress, catalase enzyme activity in visceral adipose tissue increases [56,58] or decreases significantly after administration of a high-fat diet [61]. The disparity of our results with other studies could be explained by the difference in the study population (humans vs. rats) or by the methodology used: measurement of relative gene expression rather than catalase enzyme activity. However, the intake of a high-fat diet does not influence gene expression of the enzyme during the hours of darkness. Furthermore, the administration of melatonin in the drinking water does not prevent the effects of a high-fat diet on the relative gene expression of the enzyme during the light period. These results differ from the results obtained in the hippocampus, as melatonin administration prevents the decrease in catalase activity in obese animals [51].

Concerning the degree of activity of SAT with respect to PRAT, the enzymes behave differently, especially in the dark photoperiod which coincides with the peak period of animal activity. Obesity increases the gene expression of HO1 and decreases the relative gene expression of the antioxidant enzymes SOD^Mn^, GPx and Catalase, which makes PRAT more sensitive and dynamic as a visceral fat depot than SAT, which behaves as a more static fat depot. There is evidence that lower body subcutaneous adipose tissue accumulation is protective relative to visceral. It is postulated that SAT functions as a metabolic sink with enhanced ability, relative to visceral fat, to take up lipid from circulation and store it thereby protecting insulin sensitive tissues from ectopic deposition and therefore would be less sensitive to the effects of oxidative stress [15].

A growing body of evidence suggests that metabolic disorders, characterized by adipose tissue oxidative stress, inflammation and accumulation around organs may eventually induce organ dysfunction through a direct local mechanism. Interestingly, PRAT surrounding the kidney, influences renal function and metabolism. In this regard, PRAT emerged as an independent risk factor for chronic kidney disease (CKD) and is even correlated with CVD [20] so it could be a good target for melatonin.

Surprisingly, we did not find the same effects on the relative gene expression of redox enzymes in the perirenal fat of the rat (Figure 5). This may be since the dose of melatonin used, was enough to observe metabolic prevention, as was described in previous work of our laboratory, showing that this dose of melatonin attenuated body weight increase, hyperglycemia and hyperinsulinemia, as well as the increase in mean plasma adiponectin, leptin, triglycerides, circulating low-density lipoprotein-cholesterol, total cholesterol, triglyceride concentration [16,36] and hypothalamic gene expression of signals that regulate feeding behavior [37] was not enough to exert a protective effect on the redox enzymes analyzed in this visceral adipose tissue. It is also possible that the time schedule selected for the study did not allow to see a preventive effect of melatonin on redox enzyme gene expression and it is necessary to study its effect on the daily rhythm for each enzyme over a 24-h period as we have done in previous studies [35,46].

Besides, the results indicate that melatonin, at the dose used, was able to prevent the altered biochemical pro-inflammatory profile (IL-1b, IL-6, TNF-a, IFN-c, and CRP) and anti- inflammatory profile (IL-4 and IL-10) seen in rats fed with a high-fat diet [16]. It may be interesting to indicate that in obese animals the amplitude of the nocturnal melatonin peak was markedly decreased [11], so that, the dose of melatonin used in this study perhaps is not enough to maintain the amplitude of the melatonin peak.

Furthermore, this reduction in the amplitude of the nocturnal melatonin peak observed in obese animals could be disrupting the circadian system which, as we have shown in our results, also has effects on the night/day pattern expression of the redox enzymes.

### Limitations of the Study

The study has certain limitations, since it must be taken into account that the data provided refer to relative gene expressions, which may or may be not further processed. In addition, the data presented only refer to two punctual schedules. Further work is needed to analyze the possible variations over 24 h of the gene expressions of each of the enzymes together with their respective proteins and/or enzyme activity.

## 4. Materials and Methods

### 4.1. Animals and Experimental Design

Male Wistar rats (70 days of age, 230–260 g) were maintained under standard conditions with controlled light (12:12 h light/dark schedule; with lights on at 08:00 a.m.) and temperature (22 ± 2 °C). Normal rat chow contained 3% fat, 16% protein, and 60% carbohydrate (mainly as starch with less than 0.4% fructose), providing a total caloric content of 2.9 Kcal/g. The high (35%)-fat chow contained 35% carbohydrates and 20% proteins, providing a total caloric content of 5.4 Kcal/g (Table 2).

**Figure 6:**
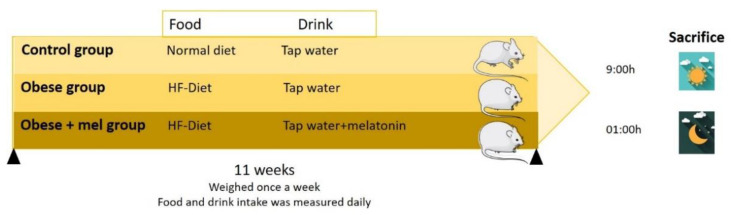
Study design, describing the food and drink administered to the animals resulting in the three different study groups: Control, obese and obese + melatonin group. Animals were sacrifice at two-time intervals: at light period 9:00 a.m. and at the middle of the scotophase (01:00 a.m.).

Animals were randomly divided into three groups (n = 15 per group) as follows: (i) control; (ii) high-fat diet (obese); (iii) high fat diet (obese) + melatonin from day one of high fat diet administration. Rats had free access to high-fat or control chow and one of the following drinking solutions for 11 weeks: (a) tap water; (b) 25 μg/mL of melatonin. Water bottles were changed every other day. Food and drink intake was measured daily. Animals were weighed once a week for 11 weeks and were euthanized by decapitation under conditions of minimal stress at two-time intervals: at light period 9:00 a.m. and at the middle of the scotophase (01:00 a.m.) (Figure 6). The melatonin dose was chosen considering that rats drank about 30 mL/day with 90–95% of this total daily water taken up during the dark period, the daily melatonin dosage used provided approximately 2.3 mg/kg melatonin. This dose is in the range of the doses used in the literature [42]. In previous studies of the group, this dose of melatonin effectively prevented the negative metabolic and inflammatory effects of obesity [16,36,37].

The subcutaneous and perirenal adipose tissue were quickly dissected and stored frozen at −80 °C until analyzed.

The experiments were approved by the Comite Ético de Experimentación Animal (CEEA, RD 53/2013) of our center and by the Comunidad Autónoma of Madrid and performed according to the European Commission Directive (2010/63/EU) for the care and management of experimental animals and complied with the ARRIVE guidelines for animal research.

### 4.2. Real-Time PCR

Total RNA extraction was performed using the RNeasy protect mini kit and was analyzed using QuantiTec SYBR green kit (Qiagen, Hielden, Germany). The iScript™ cDNA Synthesis Kit (Bio-Rad Laboratories SA, Madrid, Spain) was used to synthesize cDNA from 1 μg of total RNA, according to the manufacturer’s protocol. The house keeping gene β-actin was used as a constitutive control for normalization. Reactions were carried out in the presence of 200 nM of specific primers for Heme oxygenase 1 (HO1), Heme oxygenase 2 (HO2), Superoxide dismutase Cu/Zn (SOD Cu/Zn), Superoxide dismutase Mn (SOD Mn), Catalase (CAT), Glutathione Peroxidase (GPx) and Glutathione Reductase (GR). Primers were designed using Primer3 software (The Whitehead Institute, https://bioinfo.ut.ee/primer3-0.4.0/primer3/input.htm (accessed on 10 November 2022)) that are shown in Table 3.

PCR reactions were carried out in an Eppendorf RealPlex Mastercycler (Eppendorf AG, Hamburg, Germany). The real-time qPCR reaction program included a 94 °C enzyme activation step for 2 min followed by 40 cycles of 95 °C denaturation for 15 s, 60 °C annealing for 30 s and 72 °C extension for 30 s. Detection of fluorescent product was carried out at the end of the 72 °C extension period. Serial dilutions of cDNA from control were used to perform calibration curves in order to determine amplification efficiencies. For the primers used there were no differences between transcription efficiencies, the amount of initial cDNA in each sample being calculated by the 2^−ΔΔCt^ method [25]. All samples were analyzed in triplicate and in three different measures. The PCR device automatically calculated fractional cycle at which the amount of amplified target becomes significant (Ct).

To estimate whether treatment or time of day modified the expression of fat β-actin, PCR employing serial dilutions of this housekeeping gene was performed. In this study Ct did not vary significantly as a function of treatment or of time of day, indicating the validity to employ β-actin as a housekeeping gene.

### 4.3. Data Analysis

After verifying normality of the data distribution, the statistical analysis of the results was performed by analysis of variance (ANOVA) followed by Holm-Sidak multiple comparisons tests, as stated. The ANOVA was used to test for differences between individual data grouped according to the levels of each factor, i.e., treatment, diet, time period, fat depot and for interactions between the factors. Five hypotheses were tested in the ANOVA: (a) there was no difference between the levels of treatment (melatonin or vehicle); (b) there was no difference between the levels of diet (normal or high-fat diet); (c) there was no difference between the levels of time period (day or night); (d) there was no difference between the levels of fat depot (SAT or PRAT); (e) there was no interaction among the factors. *p* values lower than 0.05 were taken as evidence of statistical significance.

## 5. Conclusions

All the mentioned data suggest that all the enzymes studied, except for SOD Mn, GPx and Catalase, showed differential changes in their gene expression as a function of time and fat depot analyzed in the control group. The administration of a high-fat diet causes the disappearance of the temporal changes in the expression of some of the genes studied in the two fat depots analyzed, and previously described in the control group. Melatonin administration combined with a high-fat diet administration, partially prevents the effects of a high fat diet on the gene expression of the enzymes studied. Besides, our results also suggest that PRAT is more sensitive than SAT to increased oxidative stress induced by obesity. Melatonin selectively prevents the relative gene expression of redox enzymes changes in perirenal and subcutaneous adipose tissue from animals fed a high-fat diet.

## Figures and Tables

**Table 1:** Comparison of the relative gene expression of each redox enzyme between the two adipose fat depots (PRAT vs. SAT) at the same schedule. Values normalized with the 09:00 a.m. controls. Arrows indicate whether the relative gene expression of each enzyme is increased (↑) or reduced (↓) comparing same group and schedule, but different tissue.

***HO1***	**09:00 a.m**	**01:00 a.m.**
Control	−	↑ PRAT
Obese	↑ PRAT	↑ PRAT
Obese+MT	↑ PRAT	↑ PRAT
***HO2***	**09:00 a.m.**	**01:00 a.m.**
Control	**ONLY EXPRESSED IN PRAT**	
Obese		
Obese+MT		
***SODCu/Zn***	**09:00 a.m.**	**01:00 a.m.**
Control	−	−
Obese	−	−
Obese+MT	↓ PRAT	↓ PRAT
***SODMn***	**09:00 a.m.**	**01:00 a.m.**
Control	−	↑ PRAT
Obese	−	−
Obese+MT	−	↓ PRAT
***GPx***	**09:00 a.m.**	**01:00 a.m.**
Control	−	−
Obese	−	−
Obese+MT	−	↓ PRAT
***GRed***	**09:00 a.m.**	**01:00 a.m.**
Control	−	↓ PRAT
Obese	−	↓ PRAT
Obese+MT	−	−
***Catalase***	**09:00 a.m.**	**01:00 a.m.**
Control	−	↓ PRAT
Obese	−	↓ PRAT
Obese+MT	−	↓ PRAT

**Table 2:** Composition and calorie content of the diets used in the study.

	Control Diet (CD)	High Fat Diet (HFD)
% Fat	3	35
% Carbohydrates	60	35
% Proteins	16	20
% Vitamins y minerals	21	10
Caloric content (Kcal/g)	2.9	5.4

**Table 3:** Sequence of the primers used for real-time PCR.

**Gene**	**Primer**	**Gene**	**Primer**
HO1	5′-tgctcgcatgaacactctg-3′ 5′-tcctctgtcagcagtgcc-3′	GPx	5′-tgcaatcagttcggacatc-3′ 5′-cacctcgcacttctcaaaca-3′
Catalase	5′-gaatggctatggctcacaca-3′ 5′-caagtttttgatgccctggt-3′	GRed	5′-atcaaggagaagcgggatg-3′ 5′-gcgtagccgtggatgactt-3′
SOD^Cu/Zn^	5′-ggtggtccacgagaaacaag-3′ 5′-caatcacaccacaagccaag-3′	SOD^Mn^	5′-aaggagcaaggtcgcttaca-3′ 5′-acacatcaatccccagcagt-3′

## Data Availability

Data are available upon request to the authors at the Department of Biochemistry and Cell Biology, Faculty of Medicine, Universidad Complutense of Madrid.
